# The usefulness of models and simulators for training practical bovine artificial insemination skills

**DOI:** 10.3389/fvets.2023.1240978

**Published:** 2023-08-30

**Authors:** Heitor Azuaga Filho, Bruno Colaço, Rita Payan-Carreira

**Affiliations:** ^1^Instituto Federal de Educação, Ciência e Tecnologia de Mato Grosso (IFMT), Campus Professor Olegário Baldo, Cáceres, Brazil; ^2^Veterinary and Animal Research Centre (CECAV), University of Trás-os-Montes and Alto Douro (UTAD), Vila Real, Portugal; ^3^Associate Laboratory for Animal and Veterinary Sciences (AL4AnimalS), and Department of Zootechnics, University of Trás-os-Montes and Alto Douro (UTAD), Vila Real, Portugal; ^4^Comprehensive Health Research Centre (CHRC), and Department of Veterinary Medicine, Science and Technology School University of Évora, Évora, Portugal

**Keywords:** AI training, AI practice, simulators' validity, assessment, reproduction, bovine

## Introduction

Using models and simulators across medical science programs allows educators to implement learning experiences without the need to work with living beings while allowing students to practice particular procedures in safe settings and even experience error without compromising the patient's wellbeing or health. The main overall advantages of models and simulators is a reduction of animal stress during manipulation (1). They also contribute to decreasing student anxiety and improving their motivation, self-confidence, and self-efficacy (2).

For a long time, animals have been viewed as indispensable to training technical competencies in veterinary medicine students, including in the field of theriogenology. A proper training program in bovine artificial insemination (AI) includes both the development of technical skills (such as the manipulation of frozen semen) and knowledge acquisition about bovine reproductive anatomy and physiology, preparation of material, handling of semen, and the AI procedure (3). In Veterinary programs, this knowledge also encompasses breeding programs and genetic selection.

Over the years, bovine artificial insemination preparation has been based on lectures, observation of videos, and hands-on work, frequently using female genitals collected from slaughterhouses and live animals. However, more recently, concerns about animal wellbeing and the potential harm infringed to animals used in AI skills training has raised concerns in both students and teachers, even leading to divergent views about Veterinary Medicine education, especially regarding the refinement of live animal use (2, 4). These ethical concerns, combined with the shift of learning paradigms toward competency-based education, have impelled the industry to heighten standards for which models, simulators, and other alternative resources are further developed, altogether enabling students to practice and master the required skills. Most importantly, these advancements have allowed for coping with the 3Rs' recommendations for animal use in research and education.

These humane concerns assume particular relevance in the training of theriogenology-related procedures. In this field, some procedures are particularly challenging because they are performed in body structures hidden from the eye, and, therefore, they cannot be directly observed by the trainee or the educator (5). This is the case with training transrectal palpation and AI in large animals.

Nowadays, models (i.e., a static representation of anatomical structures or physiological processes) (6) and simulators (defined as devices that imitate real patients, anatomic regions, or clinical tasks) (2, 7) are used for training transrectal palpation and artificial insemination in cattle. These allow the trainee to replicate the procedures as many times as necessary for autonomy to be reached, while also providing formative assessment of each trainee's skill and warranting the “never the first time on a live animal” approach.

This article intends to revise the available information regarding the effectiveness of using models or simulators in the practical training of bovine AI procedures and to explore the assessment of their contributions to students' capacitation in bovine AI, as well as reporting on eventual gaps and providing insights on innovative features to be used in the conceptualization of future models or simulators.

## What have we now?

A snap literature review in English retrieved a few reports on the use of models or simulators in the training of bovine AI technique. *In vivo*, the two critical steps in the training of bovine AI are the confirmation of estrus (an expected training outcome in transrectal palpation) and the cervix transposition with the insemination pipette. In this article, we will focus on training cervix transposition, a critical step aimed at simulated learning of bovine artificial insemination. To train this procedure in bovine AI, models (genital tracts recovered from slaughterhouses or rubber/silicone training cervix models that can be used alone or as part of a manikin) and simulators are the most frequently used approaches.

**Figure 1:**
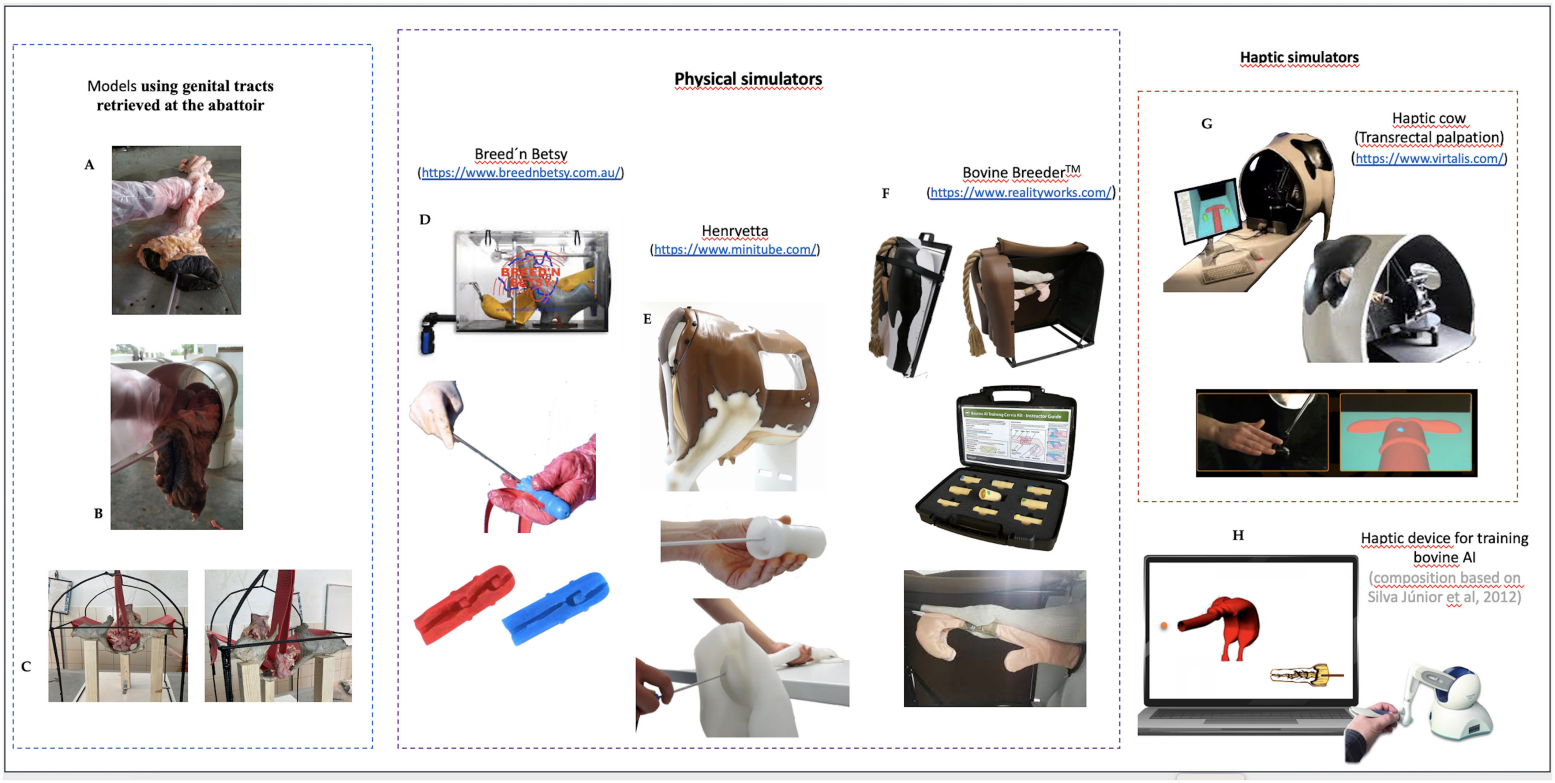
Illustration of some models and simulators available for training bovine artificial insemination. On the left side, genital tract models are used over a table **(A)** at the beginning of the training of bovine AI procedures; they can also be hidden inside a tube **(B)** or displayed on a contraption **(C)** to mimic its anatomic disposition in the pelvis. In the middle, the physical simulators: the Breed'n Betsy **(D)** uses simulated genital tracts presented inside a box, in anatomical position; others, like Henryetta **(E)**, are displayed within a standing manikin resembling a cow' rear quarter, or are collapsible and mounted over a table, like the Bovine Breeder™ **(F)**. These simulators also comprehend a movable cervix that can be used alone for training the transcervical transposition. The Haptic simulators on the right side rely on virtual reality to train transrectal palpation **(G)** or the artificial insemination technique **(H)**.

The genital tract of cows and heifers collected from slaughterhouses remains the traditional, most frequently used model for training bovine AI worldwide, promoting experience and mimicking field experience. It can be used exposed over a table at the beginning of the training (Figure 1A), allowing students to visualize the intervention and the introduction of some corrective measures to the technique, or closed within a frame that can be either a PVC rigid tube (Figure 1B) or a wooden box (8), or included in a suspension device (Figure 1C) to maintain the genital tract in a close-to-anatomic position (9). Despite these natural models being usually cheap and easily available, they also present some important disadvantages. First, they raise concerns regarding the sanitary status of the material and the possible risk of infection, particularly regarding the transmission of zoonotic diseases. Moreover, postmortem specimens lose the normal tissue tension, aggravated by the repetitive passage of the pipette across the cervical canal, leading to a facilitated passage of the pipette into the uterus after a few trials, which lowers the reliability of these models. Moreover, the material is easily perishable, which increases the demand for plenty of biological material for all the training sessions.

Synthetic models of the bovine cervix can be used for guided training at an initial hands-on stage or as a constituent of simulators (Figures 1D–F). Earlier models were made of rubber, but more recent models are produced in silicone. They usually have different dimensions, aiming to replicate the differences between heifers and cows. The main advantages of these models are their portability, the fact that the student may observe the hand positioning and motions, and the feeling of the pipette passing the cervix rings. Most synthetic cervices can be mounted on a manikin to increase the training difficulty and to allow a closer to real-life situation. Nonetheless, these models present drawbacks, including the fact that they provide experiences far from realistic due to differences in the tactile perception or elasticity of the structures mimicked, which often impair the replication of the fine hand movements associated with pipette passage across the cervix. Moreover, they are also fragile, needing replacement after a while (depending on the use and the blunt force used by the trainees).

Several commercial simulators are available to train bovine AI (Figures 1D–F). The oldest commercial manikin is the Breed'n Betsy (https://www.breednbetsy.com.au/) (Figure 1D). It comprises an interchangeable latex model of the bovine genital tract and a latex sleeve to mimic the rectum, suspended in a model of the bovine pelvis within a metal frame (10). Henryetta is a new generation of bovine AI simulators (https://www.minitube.com/catalog/en/henryetta-artificial-cow-ai-training-model-p2353/), described as a life-like, artificial cow (Figure 1E). These two are possibly the most used commercial simulators worldwide, even when others keep appearing on the market (e.g., the Ema cow or the Bovine Breeder™, Figure 1F). Compared to the genital tract model, synthetic models and simulators are usually very expensive and may not be available at many institutions. Despite having been reported to be effective in training the skills required for bovine AI [e.g., LIC training statistics presented in the MINITÜB GMBH leaflet (11)], some general difficulties of using simulators have also been evidenced when trainees change from models to live cows (10).

More sophisticated simulators include computerized elements and use virtual reality to enhance the learning experience in training bovine AI. These are called haptic simulators, and use a touch feedback source to create a computer-generated three-dimensional (3D) virtual environment or structure (12). Even though the Haptic cow (Figure 1G) has been tested within the context of bovine transrectal palpation, a similar haptic system for the training of bovine AI (Figure 1H) has yet to be tested in a group of trainees. Only a preliminary evaluation was performed by a trained veterinarian, who reported difficulties with interacting with the haptic device and the virtual environment, suggesting the need for an adaptation period before the training (12).

## Where would we like to go?

Compared with the available information on simulators for training transrectal palpation skills, far fewer outputs exist on models and simulators for training bovine AI. A key feature of simulation-based learning is ensuring that the proposed outcomes are met, skills acquisition is successful, and the trainee can transfer the new skills into practice (13) and improve their performance in the real-work task.

For transrectal palpation, simulators are considered practical and fit to effectively replace the conditions found in live animals (9, 10, 14, 15), even if their contribution to learning success remains poorly explored. There is rather more advertisement information on simulators to train AI skills than sound information on the perception (trainee point of view) and usefulness of AI skills development, autonomy, and self-confidence to further work with living cows, suggesting that there is a need to assess the advantages of each approach. Overall, the validation of available commercial simulators to train AI remains insufficient, as most results rely on the user's perception of the manikin/simulator fidelity and its utility for task learning, i.e., the subjective face validity of the instrument. Not so often, these surveys also collect the opinion of experts.

It is commonly accepted that models and simulators may not fully replicate the complexities and variations encountered when working with live animals. Therefore, often after passing an initial period working with models and simulators, trainees will spend a short period practicing AI in living cows in a herd or at abattoir facilities. However, even considering the subjective validation of simulators, we still have no answer to some questions: when are students apt to start working with living cows? At the end of the simulator training, can students transfer the skills acquired into practice without harm to the cow? What criteria should they meet to prove that the model or simulation training was successful?

Furthermore, simulators are seldom assessed for construction or concurrent validity (or transferability) (16). It is critical to determine if a given simulator can discriminate between novices and experts, if it is sensitive enough to assess the trainee's progress, and how much it contributes to a successful transition into performance of the real task.

It is our understanding that the models and simulators used should account for more than the ability to pass the cervix (yes vs. no) in the model/simulator and living animals. Considering that two key issues regarding the AI technique are the ability to pass the AI pipette throughout the cervix and the time spent in the process, it would be desirable to include the second as a criterion in assessing the trainee's proficiency. To address this purpose, it may be necessary to add sensors (electronic or electromagnetic) to physical simulators that would signal pipette transposition at crucial points in the genital tract. Virtual reality–based simulators might also benefit from the transition to a hybrid or mixed reality, the improvement of haptic interfaces, the incorporation of artificial intelligence methods to validate pipette transposition, and the correctness and economy of motions, which, taken together, could allow for determination of a task execution score.

## Brief conclusions

There is no doubt that using models and simulators at the initial stages of bovine AI training potentially reduces the harmful effects of training the AI procedures directly in a living animal, thereby contributing to the wellbeing of cows used for education. Nevertheless, it is also true that, rooted in the information provided by other fields of education using simulators and the sparse information available, new studies are needed to correctly appraise the value and usefulness of simulators available to train bovine AI. Information on efficacy models or simulators in training bovine AI skills remains insufficient to allow for careful selection of the available commercial models and simulators or to determine the most successful instructional approach. Since using non-animal devices requires educators to design and implement effective strategies to enhance the success of competency acquisition while providing practical and safe hands-on training, instructional design *per se* also affects the outcome. Therefore, this validation ought to address the entire intervention.

There are clear opportunities in this field; new developments, the improvement of existing simulators with add-ons useful for skills assessment, and the reduction of initial investment and maintenance costs should be considered to attain a broader implementation of the 3Rs.

## Author contributions

All authors listed have made a substantial, direct, and intellectual contribution to the work and approved it for publication.
